# Evaluation of ^18^F-2-deoxy-2-fluoro-glucose positron emission tomography for gastric cancer screening in asymptomatic individuals undergoing endoscopy

**DOI:** 10.1038/sj.bjc.6604062

**Published:** 2007-11-27

**Authors:** H Shoda, Y Kakugawa, D Saito, T Kozu, T Terauchi, H Daisaki, C Hamashima, Y Muramatsu, N Moriyama, H Saito

**Affiliations:** 1 Research Center for Cancer Prevention and Screening, National Cancer Center, Tsukiji 5-1-1, Chuo-ku, Tokyo 104-0045, Japan; 2 Cancer Screening Division, Tsukiji 5-1-1, Chuo-ku, Tokyo 104-0045, Japan; 3 Endoscopy Division, National Cancer Center Hospital, Tsukiji 5-1-1, Chuo-ku, Tokyo 104-0045, Japan; 4 Cancer Screening Technology Division, Tsukiji 5-1-1, Chuo-ku, Tokyo 104-0045, Japan

**Keywords:** gastric cancer, screening, endoscopy, FDG-PET, sensitivity

## Abstract

^18^F-2-deoxy-2-fluoro-glucose Positron Emission Tomography (FDG-PET) has been recently proposed as a promising cancer-screening test. However, the validity of FDG-PET in cancer screening has not been evaluated. We investigated the sensitivity of FDG-PET compared with upper gastric endoscopy in gastric cancer screening for asymptomatic individuals. A total of 2861 consecutive subjects (1600 men and 1261 women) who were asymptomatic and who underwent both FDG-PET and upper gastrointestinal endoscopy between 1 February 2004 and 31 January 2005 were included in this study. Both endoscopists and a radiologist were unaware of the results of the other diagnostic tests. The FDG-PET images were examined using criteria determined by the pattern of FDG accumulation. Sensitivity and specificity of FDG-PET were calculated compared with endoscopic diagnosis as the gold standard. Among 2861 subjects enrolled in the study, there were 20 subjects with gastric cancer, of whom 18 were T1 in depth of cancer invasion. Positive FDG-PET results were obtained only in 2 of the 20 cancer subjects. The calculated sensitivity and specificity for overall gastric cancers were 10.0% (95% confidence interval (CI): 1.2–31.7%) and 99.2% (95% CI: 98.8–99.5%), respectively. ^18^F-2-deoxy-2-fluoro-glucose Positron Emission Tomography was poorly sensitive for detection of gastric cancer in the early stages.

^18^F-2-deoxy-2-fluoro-glucose Positron Emission Tomography (FDG-PET) is a technique that reflects the changes in glucose metabolism in tumour cells, and has been widely used clinically to differentiate between benign and malignant tumours (Rigo *et al*, 1996), to assess the effectiveness of chemotherapy or radiotherapy (Kelloff *et al*, 2005), and to predict prognosis (Oshida *et al*, 1998; Oku *et al*, 2002). The potential of FDG-PET for early detection of cancer has been investigated because the test enables scanning of the whole body simultaneously and non-invasively. Because of this advantage, there has been considerable enthusiasm for PET screening in Japan (Yasuda and Ide, 2005). About 60% of facilities in Japan that are equipped with PET offer PET examinations to individuals who hope to undergo cancer screening (Yasuda and Ide, 2005).

Gastric cancer is one of the most important cancers in terms of anticancer strategy because it ranks second in cancer mortality in Japan (World Health Organization Statistics, 2006). There are many other countries with patients at high risk for gastric cancer, such as those in Central and South America, Asia, and Eastern Europe. Although gastric cancer has decreased in most of the developed countries, its prevention remains an important issue in those countries. For early detection of gastric cancer, X-ray examination with a barium meal has been employed in Japan (Fukao *et al*, 1995). Efficacy of this kind of screening program has been strongly suggested, although the studies are observational (Oshima *et al*, 1986; Fukao *et al*, 1995; Mizoue *et al*, 2003). The problem with the program is that the screening test is somewhat invasive in terms of complications such as constipation being frequently seen and mis-swallowing of barium into the trachea (Tamura *et al*, 1985; Sugahara *et al*, 1992). On the other hand, with FDG-PET, there is almost no such inconvenience for screenees. For these reasons, FDG-PET has been explored as a potential alternative to the present screening test for gastric cancer in Japan. However, the validity of FDG-PET in cancer screening remains to be evaluated. Although the sensitivity of FDG-PET for gastric cancer is reported to be from 60 to 94%, most subjects evaluated in existing reports were limited to patients with advanced gastric cancers or recurrent cancers (Yeung *et al*, 1998; De Potter *et al*, 2002; Stahl *et al*, 2003; Yoshioka *et al*, 2003; Mochiki *et al*, 2004; Chen *et al*, 2005; Yun *et al*, 2005). There has been no study to measure screening sensitivity of FDG-PET for gastric cancer in average risk individuals. Therefore, in the present study, we investigated the sensitivity of FDG-PET for gastric cancer in asymptomatic individuals who underwent FDG-PET as well as screening upper gastrointestinal endoscopy, which served as the gold standard in calculating the sensitivity of FDG-PET.

## MATERIALS AND METHODS

### Subjects and study design

The Research Center for Cancer Prevention and Screening (RCCPS), National Cancer Center (NCC), Tokyo, started the one-arm prospective cohort study designed to evaluate the efficacy of multiphasic cancer screening programs in 1 February 2004 (Hamashima *et al*, 2006). Details of the screening programs are described elsewhere (Hamashima *et al*, 2006). The screening programs consisted of upper and lower gastrointestinal endoscopy or X-ray examinations and other imaging modalities such as a chest helical CT examination. These examinations were performed during the 2-day course of the screening program. Individuals who were found to have cancer lesions were treated at the National Cancer Center Hospital. Participants were enrolled nationwide. Screenees were asymptomatic men 50 years or over and women 40 years or over who gave signed informed consent approved by the Ethics Committee for Clinical Research of the NCC. Subjects who were diagnosed as having any cancer within the past 1 year, or those who had been treated for cancer or followed-up for pre-cancerous diseases based on self-reporting were excluded. All participants responded to a questionnaire describing many issues concerning life style, family history, and previous examinations within a year (Hamashima *et al*, 2006). Individuals were to be followed up annually by a questionnaire on health status, diagnostic examinations (including results), and other relevant data.

The study population in the present study was defined as consecutive screenees who underwent both FDG-PET and gastrointestinal endoscopy between 1 February 2004 and 31 January 2005 within the screening program at the RCCPS. There were a total of 2911 individuals who underwent FDG-PET, among whom 2892 individuals, including 1626 men and 1266 women, also had gastric endoscopy and thus met the criteria for inclusion. Thirty-one individuals were excluded who had undergone gastrectomy. After excluding these subjects, the study population of 2861 participants, including 1600 men and 1261 women, was included in the analyses.

The endoscopic findings and images were examined by three skilled endoscopists (HS, YK, and TK) without any knowledge of FDG-PET findings. The FDG-PET images were examined by one expert radiologist specialising in nuclear medicine (TT), who had no information about the endoscopic findings. Findings and diagnoses were recorded separately by endoscopists and the radiologist on the electronic record system at the RCCPS to create the database of the participants. After the records were completed, findings from the two modalities were compared by either of the two investigators (HS and YM) to identify true positives and false negatives from FDG-PET results for gastric cancer based on endoscopic findings as the gold standard. Gastric cancer subjects were defined as those who were diagnosed as having gastric cancer at the time of screening or on additional endoscopy performed within 1 month after the screening.

The study protocol was approved by the Ethics Committee for Clinical Research of the NCC.

Information on cancers other than gastric cancers detected in the background population from which the present study population was drawn was described previously (Hamashima *et al*, 2006).

### ^18^F-2-deoxy-2-fluoro-glucose Positron Emission Tomography procedure

The FDG-PET images were obtained using two multi-ring PET scanners (ECAT Accel, Siemens, Knoxville, TN, USA) with a transaxial resolution of 6.2 mm at full-width half-maximum. Subjects were required to fast for at least 5 h before the PET scan. Sixty minutes after injection of 2.78 MBq kg^−1^ of FDG that was produced in our radiopharmacy, emission and transmission scans were obtained from the head to the inguinal region. A three-dimensional emission scan was acquired in eight or nine bed positions for 2 min per position, followed by a two-dimensional transmission scan for 1 min per position to correct for photon attenuation using a 68Ge/Ga rod source. Images were reconstructed iteratively (ordered-subset expectation maximisation method, two iterations, eight subsets).

The standardised uptake value (SUV) was semiquantitated in the cases with uptakes suspected of being abnormal. The SUV can be calculated as the ratio of the FDG uptake in a small region of interest (placed over the lesion in an attenuation-corrected image) to the administered activity adjusted for the body weight of the patient (Bombardieri *et al*, 2003).

### Assessment of FDG-PET findings

**Figure 1:**
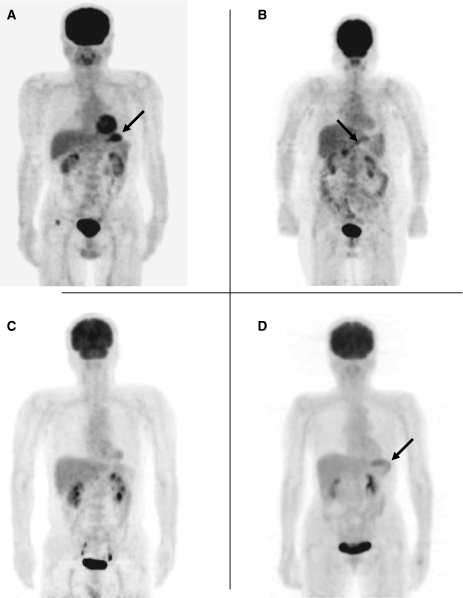
Assessment of FDG-PET findings. (**A**) PET scan demonstrates spotty or focal accumulation that is stronger than the uptake in the liver (arrow). (**B**) PET scan demonstrates focal accumulation in the area of the lower stomach (arrow). (**C**) PET scan demonstrates no definite accumulation of FDG in the stomach. (**D**) PET scan demonstrates diffuse accumulation (normal physiological accumulation) of FDG in the stomach (arrow).

Criteria for the assessment of FDG-PET findings for gastric lesions vary among facilities despite the widespread use of the guidelines for the FDG-PET procedure, mainly due to the difficulties caused by physiological uptake in the stomach. Because there are no established criteria for assessing FDG-PET findings, we determined the following criteria based on previous reports (Cook *et al*, 1996; Gordon *et al*, 1997; Shreve *et al*, 1999; Koga *et al*, 2003): (1) positive pattern 1 – spotty or focal accumulation that was stronger than the uptake in the liver (Figure 1A); positive pattern 2 – any accumulation in the area of the lower stomach (Figure 1B). This category was based on a report by Koga *et al* (2003), suggesting that physiological gastric FDG uptake is significantly higher at the oral end than the anal end, and that a stronger gastric FDG uptake at the anal end might therefore be suggestive of a pathological uptake. (2) negative pattern 1 – no definite accumulation in the stomach (Figure 1C); negative pattern 2 – diffuse accumulation in the stomach, considered to be a normal physiological uptake (Figure 1D). The judgment of FDG-PET accumulation was made based only on PET without CT scan. Positive whole body FDG-PET findings were obtained in 9% of 2911 subjects who had FDG-PET examinations. Approximately one-fourth of those with positive FDG-PET required further investigation in addition to the examinations included in the screening program. Detailed information will be described elsewhere.

### Upper gastrointestinal endoscopy

All subjects were administered a 100 ml solution containing 1 g of Pronase and 1 g of sodium bicarbonate to remove mucus and bubbles on the gastric mucosa before examination. The antiperistaltic (20 mg of scopolamine butylbromide or 1 mg of glucagon) and sedative (17.5–35 mg of pethidine hydrochloride or 2–10 mg of midazolam) agents were injected intravenously except when they were contraindicated. We used standard commercial video endoscopic equipment (GIF TYPE H-260 or Q260; Olympus Co., Tokyo, Japan). Endoscopic images were obtained and recorded in a standardised pattern, which covered the entire gastric mucosa in about 50 shots. We added chromoendoscopy with 0.2% solution of indigo-carmine in all subjects after conventional observation. All lesions that appeared potentially malignant were biopsied for histopathological examination. The location, description of lesions, and diagnosis were recorded just after the gastrointestinal endoscopy. Size of cancer lesions was measured on the surgically or endoscopically resected specimen. Endoscopic images were reviewed primarily on the same day by three endoscopists (HS, YK, and TK) to determine whether there were any lesions overlooked during endoscopy. If any suspicious findings were suggested to have been overlooked, the screenees were recommended to undergo an additional endoscopy.

### Histopathological findings

The final pathological diagnosis was confirmed from specimens resected surgically or endoscopically. The depth of cancer invasion was recorded according to the TNM clinical classification (Sobin and Wittekind, 1997). Two pathologists interpreted the histopathologic features and when there was a disagreement, a senior pathologist reviewed the features to resolve the disagreement.

### Statistical analyses

The Student's *t*-test was used to assess the difference in the mean age between gastric cancer subjects and those without gastric cancer or between male and female subjects. Statistical significance for comparison of items other than age between subjects with gastric cancer and subjects without gastric cancer was assessed by *χ*^2^ test. The difference in SUV between true positives and false positives was also analysed by the Student's *t*-test. *P*-values <0.05 were considered statistically significant and 95% confidence intervals (CIs) were calculated based on a binominal distribution.

## RESULTS

The characteristics of the subjects enrolled in the study are shown in Table 1. Among 2861 subjects enrolled in the study, gastric cancers were detected by gastrointestinal endoscopy in 20 subjects, including 18 men and 2 women. The mean age of all subjects was 59.8 years old, and there was no statistically significant difference between subjects with gastric cancer and subjects without gastric cancer. Males were older than females both among subjects with gastric cancer and subjects without gastric cancer. The proportion of males to females was significantly higher for subjects with gastric cancer than for subjects without gastric cancer (Table 1). There was no significant difference in the frequency of family history of gastric or any other cancer, or of previous examinations between subjects with gastric cancer and subjects without gastric cancer (Table 1).

Detailed clinical features of gastric cancers detected by endoscopy are shown in the bottom of Table 1. Histopathologically, about half of the cancers were well or moderately differentiated adenocarcinoma. Of the 20 gastric cancers, 18 were of T1 stage (Table 2), among which cancer invasion into the gastric wall was confined to the mucosa in 12 subjects, and to the submucosa in six subjects. Only two subjects among 20 cases with gastric cancer showed positive results with PET. The first patient had T4 cancer (Borrmann type 2, poorly differentiated adenocarcinoma), and the FDG-PET showed strong and focal accumulation in the area of the upper gastric body as ‘positive pattern 1’. The second patient had T1 cancer (a superficial depressed type, signet ring cell carcinoma), and the FDG-PET showed stronger accumulation in the area of the lower gastric body, which was clearly judged as ‘positive pattern 2’.

The overall sensitivity, specificity, and positive predictive values were 10.0% (95% CI: 1.2–31.7%), 99.2% (95% CI: 98.8–99.5%), and 8.3% (95% CI: 1.0–27.0%), respectively, and the negative predictive value was 99.4% (95% CI: 99.0–99.6%) (Table 3). There were 22 subjects with positive FDG- PET accumulation in addition to two cases of gastric cancer. These 22 subjects had no other neoplastic lesions detected in the colon, nor in the other abdominal organs by colonoscopy and ultrasound sonography.

We compared the SUV between FDG-PET true positives (two subjects) and FDG-PET false positives (22 subjects). The mean±s.d. of the SUVs was 4.9±1.46 in true positives and 4.5±0.96 in false positives, and there was no significant difference between them.

## DISCUSSION

We have shown that the sensitivity of FDG-PET for gastric cancer is as low as 10% in this study. Although the sensitivity of FDG-PET for gastric cancer has been reported in some studies to range from 60 to 94% (Yeung *et al*, 1998; De Potter *et al*, 2002; Stahl *et al*, 2003; Yoshioka *et al*, 2003; Mochiki *et al*, 2004; Chen *et al*, 2005; Yun *et al*, 2005), the subjects used in those reports were primarily clinically diagnosed, preoperative, advanced cancer, or recurrent cancer cases, and thus the sensitivity values calculated in those studies may not represent screening sensitivity. Screening sensitivity can only be measured in an asymptomatic population, preferably by performing diagnostic examination such as endoscopy on all subjects in order to identify cancer subjects in the population. There have been no other studies that have evaluated the sensitivity of FDG-PET for gastric cancer in an asymptomatic population based on the findings of endoscopy as the gold standard.

There are a few issues to be addressed, which might have influenced the sensitivity calculated in this study. Firstly, our case series of screen-detected cancers consists largely of cancers in the early stages, and the proportion of more advanced cancers was very small (2 of 20) (Table 2). Our previous report showed a little higher detection rate of gastric cancer in men than expected, which suggested possible overdiagnosis among screen-detected cancers (Hamashima *et al*, 2006). The high proportion of early cancers, including those of overdiagnosis among screen-detected cancers, could be a reason for our low sensitivity. There is one study from Japan in which the sensitivity of FDG-PET for early gastric cancer could be calculated, although the subjects used were clinically diagnosed cancers. Mochiki *et al* (2004) reported that the sensitivity was 40% in gastric cancers of T1 stage subsequently treated surgically. Although detailed information on the depth of cancer invasion was not available in that paper, the case series in their report was estimated to be of a more invasive nature than those in the present study in terms of depth of invasion. Because the indication for surgical resection of gastric cancer in terms of depth of cancer invasion is submucosal or deeper invasion in Japan, the subjects with T1 stage cancers would have had submucosal invasion in their study. In the present study, 12 out of 18 T1 cancers were intramucosal cancer, which did not necessarily require surgery. This difference might explain the difference in sensitivity for early cancer detection between the two studies. However, even when intramucosal cancers were excluded from the calculation, the sensitivity was only 12.5% (one positive out of eight). Secondly, in our study, we performed chromoendoscopy on all screenees, which might have enhanced the ability to detect small cancer lesions. Thirty percent of cancer lesions were 10 mm or less in diameter (Table 1). In any case, the calculated sensitivity in this study might be underestimated due to potential overdiagnosis relevant to screen-detected cancer as mentioned above.

The FDG-PET procedure employed in this study is based on the standard method used in clinical practice, except for the criteria for assessment of cancer. PET findings were assessed according to the criteria, which we defined, due to lack of established criteria. The main difficulty in FDG-PET diagnosis of stomach cancer is physiological uptake in the stomach (Cook *et al*, 1996; Gordon *et al*, 1997; Shreve *et al*, 1999; Koga *et al*, 2003), but there was no cancer subject in whom we had difficulty in differentiating physiological uptake from cancer lesions. Nevertheless, it is possible that there were tiny cancers overlooked due to significant FDG background uptake. As physiological uptake is more significant in the oral end of the stomach than in the anal end, screen-detected cancers with FDG-PET might be biased towards cancers in the anal end of the stomach.

In this study, there were 22 subjects with false-positive PET. There remains the possibility that upper gastrointestinal endoscopy had overlooked tiny lesions rather than that they were false positives. However, endoscopic images recorded in as many as approximately 50 shots were reviewed just after endoscopy to check for overlooked lesions. Therefore, it is unlikely that overlooked lesions were a main reason for such a low sensitivity.

It might be necessary to compare FDG-PET findings with those of existing examinations, such as barium meal and gastrointestinal endoscopy in terms of efficacy, cost, convenience, and radiation dose. Efficacy has been evaluated only for barium meal examinations in Japan by case–control studies (Oshima *et al*, 1986; Fukao *et al*, 1995; Mizoue *et al*, 2003). ^18^F-2-deoxy-2-fluoro-glucose Positron Emission Tomography is more expensive than the other two procedures (85 000 Japanese yen or 772 US$ for FDG-PET, 12 680 yen or 115 US$ for endoscopy in our screening program, and about 82 US$ for barium meal examination). There is much less inconvenience for screenees with FDG-PET than is seen after endoscopy or barium meal examination, which are often accompanied by discomfort during examination, side effects of antispasmodic agents, or constipation after examination. With regard to radiation dose, the average dose at our facility during the current study was 3.2 mSv for FDG-PET and 4.4 mSv for CT, which are similar to prior reports of barium meal examination that ranged from 3.0 to 9.3 mSv (Broadhead *et al*, 1995; Geleijns *et al*, 1998), although the radiation dose of screening fluorography in Japan would be lower than barium meal examination as a diagnostic test (Kato *et al*, 1999).

This study did not evaluate the efficacy of FDG-PET screening for gastric cancer. Moreover, in this study, the sensitivity for more advanced cancers, which would be less likely to be affected by overdiagnosis, could not be measured due to an insufficient number of such cancers among screen-detected cancers. The sensitivity calculated here might thus be an underestimate of that for all gastric cancers. However, in conclusion, it was clearly demonstrated in this study that FDG-PET is poorly sensitive for the detection of gastric cancer in the early stages.

## Figures and Tables

**Table 1:** Characteristics of subjects enrolled in this study

**Variables**	**Subjects with gastric cancers (*n*=20)**	**Subjects without gastric cancers (*n*=2841)**	***P*-value^§^**
*Age (mean±s.d.) (year)*			
Overall	63.1±5.1	59.8±7.0	0.0368
Male	64.1±4.1	61.1±6.0	0.0330
Female	53.5±0.7	58.2±7.7	0.3919
			
*Sex*			
Male/female	18/2	1582/1259	0.0043
			
*Family history of gastric cancer*			
Within second degree family	6	591	0.4638
Within first degree family	5	470	0.4769
			
*Family history of any cancer*			
Within second degree family	14	1842	0.8048
Within first degree family	11	1511	>0.9999
			
*History of gastric examinations* a	11	1578	>0.9999
Barium meal X-ray examination	8	1051	0.9640
Gastrointestinal endoscopy	4	780	0.6217
			
*Characteristics of gastric cancer*			
Location^b^ (U area/M area/L area)	4/5/11		
Size^c^ (–10 mm/11–20 mm/21 mm–)	6/7/7		
Histological type			
Differentiated adenocarcinoma (Well/Mod)	11(11/0)		
Undifferentiated adenocarcinoma (Por/Sig/Mixed (Sig/Por))	9(1/4/4)		

Mod=moderately differentiated adenocarcinoma; Por=poorly differentiated adenocarcinoma; Sig=signet ring cell carcinoma; Well=well-differentiated adenocarcinoma.

^§^Statistical significance for comparison of each item between subjects with gastric cancer and without gastric cancer.

a Proportion of subjects who had undergone stomach examination as a screening test or diagnostic test with X-ray examination and/or gastrointestinal endoscopy within 1 year before the screening endoscopy in this study.

b Location of a lesion is based on the ‘Japanese Classification of Gastric Carcinoma’ (The 13th Edition, 1999) by Japanese Gastric Cancer Association.

c Maximum diameter of cancer lesions.

**Table 2:** FDG-PET results according to depth of cancer invasion

		**Depth of invasion^a^**			
		**T1**	**T2**	**T3**	**T4**
FDG-PET positive	*n*=2	1	0	0	1
FDG-PET negative	*n*=18	17	1	0	0
Total	*n*=20	18	1	0	1

FDG-PET denotes ^18^F-2-deoxy-2-fluoro-glucose positron emission tomography.

T1: tumour invades lamina propria or submucosa.

T2: tumour invades muscularis propria or subserosa.

T3: tumour penetrates serosa (visceral peritoneum) without invasion of adjacent structures.

T4: tumour invades adjacent structures.

a The depths of cancer invasion were based on the TNM classification.

**Table 3:** Sensitivity and specificity of FDG-PET for gastric cancer

	**Subjects with gastric cancers (*n*=20)**	**Subjects without gastric cancers (*n*=2841)**
FDG-PET positive *n*=24	2	22
FDG-PET negative *n*=2837	18	2819

CI=confidence interval.

Sensitivity (95% CI)=2/20=10% (1.2–31.7%).

Specificity (95% CI)=2819/2841=99.2% (98.8–99.5%). Positive predictive value=2/24=8.3% (1.0–27.0%). Negative predictive value=2819/2837=99.4% (99.0–99.6%).
